# Better sanitation, with communities taking the lead

**Published:** 2013

**Authors:** Muhammod Abdus Sabur

**Affiliations:** Freelance public health consultant and former country representative: WaterAid Bangladesh.

**Figure:**
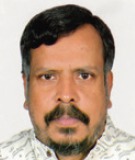
Muhammod Abdus Sabur

Most traditional sanitation programmes provide some form of subsidy to individual families to reduce the cost of building a toilet. This approach is based on the assumption that both the construction and use of toilets depend on private decisions and on hygiene behaviour at the household level. However, this approach normally results in small, step-by-step changes in sanitation coverage, with further improvements becoming steadily more difficult once early adopters and households with higher incomes have installed sanitation facilities. Few large-scale sanitation programmes of this type have been successful. It may be that households who build toilets under such heavily-subsidised programmes feel less ownership for their facilities and are therefore less inclined to make any lasting improvements to their hygiene behaviour.

## Community-led total sanitation: a new approach

Over the last 15 years, non-governmental organisations (NGOs) in Bangladesh have pioneered a new approach to sanitation development known as community-led total sanitation (CLTS). This approach has also been replicated in a number of other countries in Asia and Africa. The key difference between previous approaches and CLTS was demand creation within communities.

This approach recognises that sanitation is both a public and a private good, and that individual hygiene behaviour can affect the whole community: if your neighbours defecate in the open, then your children risk excreta-related disease even when the members of your own household use a sanitary toilet, wash their hands, and practice good hygiene. In this sense, *total sanitation* refers to a total stop on open defecation, which requires that everyone in the community either owns or has access to a sanitary toilet.

The main advantage of the total sanitation approach over conventional policies is that it is a community-wide approach. It requires that every household in the community stops open defecation and uses a sanitary toilet. This approach involves even the poorest and most vulnerable households and ensures that the community and local government focus on helping these households gain access to a sanitary toilet with a safe excreta disposal system. This process is the reverse of most conventional sanitation programmes, which tend to favour those who can afford toilets, have land available to build toilets, and are first on the list for subsidised facilities.

**Figure 1.**
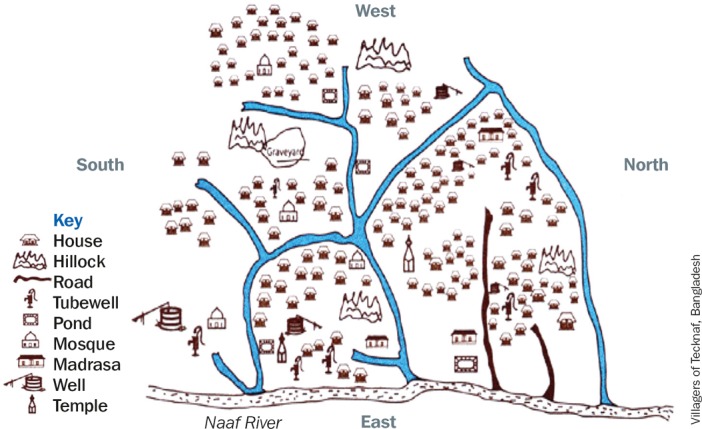
Community social mapping

## How to make it work

The community-led total sanitation approach encourages people to think about total sanitation and to consider what their communities will be like when it is achieved. It is based on the assumption that the community has the strength and willingness to overcome their problems. It recognises that outsiders may be needed to help the community identity their current situation and the need for improvement; however, the community must want to change. Therefore the role of NGO is that of a facilitator enabling communities to:

- analyse their current situation
- identity areas for improvement
- plan how to improve these areas, including training local artisans in low-cost latrine construction
- implement solutions to meet their own needs.

The NGO therefore concentrates on social development using a process of institution building and community empowerment rather than on the delivery of water and sanitation services. The process of achieving total sanitation in any geographical/administrative area starts at community level, where the community may be a village or sub-village. The important thing is that the people see themselves as a community whose members affect and support each other. As individual communities within a geographical/administrative area become motivated, neighbouring communities become aware of the improved situation and are motivated to find out about total sanitation. Eventually all communities of the geographical/ administrative area are involved and the process to achieve total coverage is in progress.

The success of the approach depends on the level of involvement of individuals within the communities. Initially, the input of the external facilitators may be high as they work to encourage members of the community to get involved. As the process develops, the need for external facilitation will decrease and eventually end. A range of participatory rural appraisal tools are used during the process, for example community mapping (see Figure 1) as the key is to help the community identity and analyse their current situation.

## Conclusion

Non-governmental organisations in Bangladesh and other countries report that they have used the total sanitation approach in stopping open defecation, using participatory techniques to raise awareness of local sanitation issues and to assist communities to solve their own problems. The combination of internal community pressure and external NGO support is reported to have enabled thousands of communities to reach total sanitation coverage without any hardware subsidies at household level.

